# Validity and Usage of the Seasonal Pattern Assessment Questionnaire (SPAQ) in a French Population of Patients with Depression, Bipolar Disorders and Controls

**DOI:** 10.3390/jcm10091897

**Published:** 2021-04-27

**Authors:** Eve Reynaud, Fabrice Berna, Emmanuel Haffen, Luisa Weiner, Julia Maruani, Michel Lejoyeux, Carmen M. Schroder, Patrice Bourgin, Pierre A. Geoffroy

**Affiliations:** 1 CNRS UPR 3212, Institute for Cellular and Integrative Neurosciences, 67000 Strasbourg, France; schroderc@unistra.fr (C.M.S.); pbourgin@unistra.fr (P.B.); 2 Psychiatry Department, Hôpitaux Universitaires de Strasbourg, 67000 Strasbourg, France; fabrice.berna@chru-strasbourg.fr (F.B.); luisa.weiner@chru-strasbourg.fr (L.W.); 3 INSERM 1114, Université de Strasbourg, 67000 Strasbourg, France; 4 Department of Adult Psychiatry, CHU de Besançon, EA 481, 25000 Besançon, France; emmanuel.haffen@univ-fcomte.fr; 5 CIC 1431 INSERM, Université de Franche-Comté, 25000 Besançon, France; 6 Laboratoire de Psychologie des Cognitions, Université de Strasbourg, 67000 Strasbourg, France; 7 Department of Psychiatry and Addictive Medicine, Assistance Publique-Hôpitaux de Paris (AP-HP), University Hospital Bichat, 46 rue Henri Huchard, 75018 Paris, France; julia.maruani@aphp.fr (J.M.); michel.lejoyeux@aphp.fr (M.L.); 8 NeuroDiderot, INSERM UMR 1141, Université de Paris, 75019 Paris, France; 9 UFR de Medicine, Université de Paris, 5 Rue Thomas Mann, 75013 Paris, France; 10 GHU Paris-Psychiatry & Neurosciences, 75014 Paris, France; 11 Sleep Disorders Center & CIRCSom (International Research Center for ChronoSomnology), Strasbourg University Hospital, 67000 Strasbourg, France; 12 Department of Child and Adolescent Psychiatry, Strasbourg University Hospital, 67000 Strasbourg, France

**Keywords:** seasonal affective disorder, circadian rhythms, bipolar disorder, psychometry

## Abstract

The Seasonal Pattern Assessment Questionnaire (SPAQ), by Rosenthal et al. (1984), is by far the most used questionnaire to evaluate seasonal effects on mood and behavior. It includes a general seasonality score (GSS), composed of 6 items, from which cutoffs have been established to screen for seasonal affective disorder (SAD). However, it has never been validated in French and associations with circadian rhythm and symptoms of depression and bipolarity remain unclear. In this study, including 165 subjects (95 controls and 70 patients with depression or bipolar disorder), we confirmed the validity of the French version of the SPAQ, with a two-factor structure (a psychological factor: energy, mood, social activity and sleep length; and a food factor: weight and appetite) and a good fit was observed by all indicators. Mood and social activity dimensions were significantly affected by seasons in the depressed/bipolar group and a stronger global seasonality score (GSS) was associated with more severe phenotypes of depression and mania. Subjects meeting SAD and subsyndromal-SAD criteria also showed a delayed circadian rhythm compared to controls. Simple tools, such as the SPAQ, can aid the identification of significant seasonal changes and have direct implications on therapeutics including the use of bright light therapy in order to enhance personalized treatments, but also to prevent adverse seasonal effects.

## 1. Introduction

Seasonal change in mood and behavior is observed in the general population [1,2,3,4,5,6], and seasonal affective disorder (SAD) can be understood as the extreme pathological end of this seasonality continuum [7,8,9]. Albeit the apparent dimensional features of seasonality, SAD has been defined as a transdiagnostic disorder that may affect patients with unipolar and bipolar disorders and corresponds to the seasonal pattern (SP) of recurrent depressive episodes occurring during the same time of the year, usually in autumn or winter with spontaneous remission in the spring or summer [10]. Other categorical approaches are proposed such as in the DSM-5 (Diagnostic and Statistical Manual of Mental Disorders, Fifth Edition, American Psychiatric Association) [11] where SP is treated as a specifier that can be applied to depressive or manic episodes, but do not define a disorder per se. Diagnostic criteria of SP include two consecutive recurrences of a major depressive or manic episode at a particular time of year, followed by a full remission or a change from major depression to mania or hypomania, or vice-versa [11]. In studies using these strict DSM-5 diagnostic criteria, reported prevalences of SAD range from around 1% to 3% in North America and Europe (for review: Byrne et al., 2008 [12]) with higher prevalences at higher latitude [13], while prevalences close to 10% have been reported when using less stringent definitions of SAD [14]. Prevalence of SAD decreases with age, and higher prevalences of SAD have been reported in women [15]. SAD is overrepresented in patients with bipolar disorders (BD), it is estimated that a quarter of patients with BD present an SP in their depressive episodes and a sixth in their manic episodes [8,16].

Current knowledge on the etiology of SAD suggests the implication of several biologic mechanisms (for review: Maruani et al., 2018 [17]), including genetic variants in circadian clock genes [18,19,20,21], circadian alterations such as phase shift [22], abnormal melatonin secretions [22,23] and alterations of the serotoninergic system [24,25]. Bright light-therapy is endorsed as the first line treatment of SAD in multiple guidelines, with meta-analyses and randomized control trials attesting to its efficiency [26,27,28,29,30]. SAD has been described as a significant mental health problem and a public health challenge, given its high prevalence, the recurrence and duration of the episodes as well as the associated impairments [31]. Patients suffering from SAD can experience depressive symptoms during 40% of the year with considerable socio-professional and economic impact [32]. SAD is also associated with increased severity of BD [16] and increased cardiovascular risk factors [33].

The Seasonal Pattern Assessment Questionnaire (SPAQ), designed by Rosenthal and coworkers [34], is by far the most used questionnaire for SAD. The SPAQ can be used both in the general population and in individuals with mood disorders, due to the absence of assumptions about the direction of seasonal change and the ability of this instrument to measure seasonal change independently of illness [8]. Kasper et al. [1] have proposed a cut-off to screen for SAD using the SPAQ. However, there are inconsistent results regarding the psychometric properties of this cut off, with reported sensitivity ranging from 40% [35] to 94% [36]. It should be noted that the SPAQ was not initially designed to be used as a dichotomous measure, it includes dimensional aspects of SP which are currently under used. Yet dimensional aspects of SP probably reflect the underlying biological processes, as a continuum of seasonal effects’ severity [17]. Finally, the SPAQ has never been validated in French.

The primary aim of our study was (i) to validate the structure of the French version of the SPAQ, (ii) to assess the monthly pattern of the six dimensions of the SPAQ—namely sleep length, social activities, mood, energy level, weight and appetite; and (iii) to investigate the association between the degree of seasonality and circadian rhythms, quality of life and diverse symptoms of depression and bipolarity (anxiety, depressive and manic symptoms). We hypothesize that the confirmatory factor analysis will allow validation of the two factor structure proposed by Mersch et al. [35], that most dimensions will follow a seasonal pattern and that the stronger the seasonal pattern the stronger the associated symptoms.

## 2. Materials and Methods

### 2.1. Population

Participants were recruited throughout the year through social media and the FondaMental Foundation (French network of expert centers for psychiatric disorders). The study took the form of an anonymous survey built with the QuestBack platform and an encrypted server to ensure data safety protection, it was approved by the University of Strasbourg ethics committee (unistra/cer/2020-16). An introductory text explained the study to all potential participants, and if they agreed to participate, they were asked to fill a 10-minute online self-administered questionnaire. The diagnosis of the disorder was retained if the patient declared that it was made by a physician or treated by a psychotropic medication recommended for the self-declared disorder. Inclusion criteria were that participants had to be older than 18 years and to speak French. There were no exclusion criteria based on psychiatric disorder.

The study included 165 subjects (composing the “full sample”), with 95 declaring no psychiatric disorder, defined as the “control group” and 70 patients diagnosed with depression or bipolar disorder, defined as the “depressed/bipolar” group. The average age was 37.94 (SD 11.18) years with 72.1% being women (*n* = 119). As sex and age differences were observed between the groups, subjects were matched based on these factors in analysis comparing both groups (described in the statistical analyses section). This matched sample, included 64 patients and 64 controls, with an average age of 40.55 years (SD 10.98) and 78.1% being women (*n* = 100).

### 2.2. Measures

#### 2.2.1. SPAQ

The SPAQ is composed of several sections. Basic information on demographic data (10 items) is followed by the evaluation of the seasonal change across 6 dimensions, namely sleep length, social activity, mood, weight, appetite and energy level. The degree of change of the 6 dimensions are assessed by a single item each scored from 0 (no change) to 4 (extremely marked change). The 6 scores are summed up to calculate the general seasonality score (GSS), which thus ranges from 0 (no seasonality) to 24 (extreme seasonality).

Then, in a calendar section, the subject indicates which months he/she sleeps the most and least, has the most and least social activities, feels the best and worst, gains and loses the most weight, eats the most and least (10 items). Thus, a positive and a negative calendar item corresponds to each of the first five dimensions. Subsequently, the subject specifies how much his/her weight varies during the year (in kg for the French version and lbs. for the English version), the duration of sleep for each season (in hours), and if he/she experiences a modification of food preferences according to season (yes/no, and if yes, then an open comment section). Finally, the subject has to state if he/she feels that these seasonal changes are a problem for him/her (yes/no, if yes, then a 5-item Likert scale ranging from mild to disabling is proposed). In the present study, the SPAQ was translated from English to French by French-native speakers, EH and PAG, and back-translated by an English native speaker, Sarah King. The translation and the original English questionnaire are available in Supplementary data, File S1.

Generally, only the GSS is used for research purposes, and the calendar item “feel worst” is sometimes used to qualify the seasonality as being either winter or summer. Kasper et al. [1] have also proposed screening criteria for SAD based on the combination of 3 factors: (1) a GSS greater or equal to 11, (2) a seasonal change declared as being a moderate problem or worse (score of 2 or more out of 5), (3) a declared month of “feeling worst” in either January, February or both, for winter SAD, and either July, August or both, for summer SAD. They also defined criteria for subsyndromal-SAD (S-SAD), where either the GSS score is greater or equal to 11 (as for SAD) but the seasonal change is not considered a problem or only a mild problem (score of 0 or 1 out of 5); or the GSS score is of 9 or 10 (regardless of whether the seasonal change is a problem or not). The factor structure of the GSS was validated by Mersch et al. [35] indicating a good internal consistency of a two factor structure: a psychological factor (energy, mood, social activity and sleep length) and a food factor (weight and appetite). The authors also reported that the SAD criterion of the SPAQ had good specificity (94%), but low sensitivity (44%).

#### 2.2.2. Subsidiary Measures

Mania was assessed using the Altman self-rating mania scale (ASRM) [37], composed of 5 items. The total score ranges from 0 to 20, with a higher score indicating increased severity of manic symptoms. The ASRM’s internal consistency as measured by Cronbach’s alpha is 0.75 [38].

Depressive symptoms were assessed with the Quick Inventory of Depressive Symptomatology (QIDS-16) [39], a self-reported 16-item questionnaire. The total score ranges from 0 to 27, with a higher score indicating a higher severity of depression. The QIDS-16 shows high internal consistency (Cronbach’s alpha 0.86) [39].

The EQ-5D (European Quality of life-5 Dimension) [40] is a self-rated questionnaire evaluating perceived health according to a visual analogue scale on general current health and 5 dimensions: mobility, self-care, usual activities, pain/discomfort and anxiety/depression. General health is rated from 0 to 100 with 100 corresponding to “the best health you can imagine”, and the 5 dimensions each range from 1 to 5 with higher scores indicating decreased perceived health. Reported intraclass correlations range from good to excellent [41].

Circadian rhythm parameters were subjectively assessed by two validated questionnaires. The Composite Scale of Morningness (CSM) [42] is composed of 13 items indicating the circadian preference. The total score ranges from 13 to 55, with a lower score indicating an evening type and a higher score a morning type. The Cronbach’s alpha is 0.82. The circadian type inventory (CTI) [43] is composed of 5 items evaluating if the subject is rather flexible or rigid when facing an unusual schedule (scores range from 5 to 25 with a higher score for this factor indicating a more flexible type, Cronbach’s alpha of 0.79), and of 6 items evaluating in the same situation if the subject is rather languid or vigorous (score ranging from 6 to 30 with a higher score indicating a more languid type, Cronbach’s alpha of 0.72).

### 2.3. Statistical Analyses

Analyses were conducted using R software [44] version 4.0.0. The confirmatory factor analysis was conducted on the full sample. For all other analyses, the control group was compared to the group of people previously diagnosed with BD or depression. As sex and age differences were observed between the groups, subjects were matched based on these factors, reducing the sample to 64 controls and 64 patients.

#### 2.3.1. Confirmatory Factor Analyses

The first part of the analyses consisted of confirming the factor structure of the general seasonality score (GSS) as described by Mersch et al. [35], using a confirmatory factor analysis on the full sample. In accordance with recommendations [45,46], the retained measures of fit and recommended cutoffs to confirm the model were the chi-square (the null hypothesis is “the model fits perfectly”, thus a *p*-value greater than 0.05 is sought), the Tucker–Lewis index (TLI, also called the non-normed fit index (NNFI), >0.95), the comparative fit index (CFI, >0.95), the root mean square error of approximation (RMSEA, <0.07) and the standardized root mean square residual (SRMR, <0.08). Additionally, a stratified Cronbach’s alpha was conducted to assess reliability (>0.7).

#### 2.3.2. Month by Month Variation of the GSS Dimensions

In addition, we replicated analyses by Thompson et al. [47] to separately assess the seasonality score per month by dimensions. The method consists of using the positive and negative calendar item pairs and to weight them by the corresponding dimensions of seasonal change. When both items are scored as present or when none are scored as present, the seasonality score equals zero (this dimension is neither negative nor positive for this month). When the positive item is scored as present but not the other, the seasonality score is +1 × seasonal change. Inversely, when the negative item is scored as present but not the other, the score is −1 × seasonal change. A higher score thus indicates higher mood, social activity, weight gain, more appetite and more sleep for a given month, relatively to other months. In other words:

(1) $$\begin{array}{cc} {\mathrm{Seasonality}\;\mathrm{Score}}_{\mathrm{month}\;\mathrm{and}\;\mathrm{dimension}} & \\ & =({\mathrm{positive}\;\mathrm{item}}_{\mathrm{month}\;\mathrm{and}\;\mathrm{dimensions}}\\ & -{\mathrm{negative}\;\mathrm{item}}_{\mathrm{month}\;\mathrm{and}\;\mathrm{dimenssion}})\times \left({\mathrm{degree}\;\mathrm{of}\;\mathrm{change}}_{\mathrm{dimension}}\right) \end{array}$$

We used five mixed models for repeated measures (twelve measures, one per month) to separately test the seasonality of mood, social activity, weight, appetite and sleep length, according to groups (control vs. depressed/bipolar). The inter-individual factor was the seasonality score, and the intra-individual factors were the different months.

#### 2.3.3. Association between GSS, Questionnaires and Diagnosis 

We then studied the association between GSS, questionnaires and diagnosis. As most scales did not follow a normal distribution, non-parametric tests were conducted. Kendall rank correlation coefficient (Kendall’s τ) was used to study the correlation between the GSS and other scales, and the difference in GSS by diagnostic group was assessed with the Mann–Whitney U test.

#### 2.3.4. Association between Diagnosis and Type of Seasonality 

Lastly, using chi-square analysis we studied the association between diagnosis and the type of seasonality, in terms of seasonality being a problem or not (question directly included in the SPAQ), and in terms of season. To define the season, we used the “feeling worst” and “feeling best” calendar items. The “feeling worst” seasonality was defined as being winter if the “feeling worst” item of the calendar section of the SPAQ was selected for at least one month from November to February, and no month from June to August. Inversely, the seasonality was defined as being summer if the “feeling worst” item of the calendar section of the SPAQ was selected for at least one month from June to August, and no month from November to February. Seasonality was defined as “none” if the patient declared that there was no month in particular where he/she felt worse, and it was defined as missing data when the patient declared certain months to be worse than others, but the pattern of selected months did not correspond to a winter or summer seasonality. The “feeling best” seasonality was defined similarly.

## 3. Results

### 3.1. Confirmatory Factor Analysis of the GSS

All GSS dimensions were correlated (Table 1). The confirmatory factor analysis of the two-factor structure of the GSS (psychological factors, i.e., energy, mood, social activity and sleep length; and food factors, i.e., weight and appetite) showed a very good fit, with a X^2^ of 12.22 (*p* = 0.14), a CFI of 0.988, a TLI of 0.978, an SMR of 0.035 and an RMSEA of 0.057. Stratified Cronbach’s alpha was 0.86. Thus, all fit measures corresponded to the recommended cutoffs, and the model showed high factor loading (Table 2 and Figure S1). The factors covariance was 0.55 (*p* < 0.001).

### 3.2. Association between GSS, Questionnaires and Diagnosis 

The correlation between the different GSS dimensions, namely, psychological factor, food factor and total score, with the questionnaires assessing circadian rhythms, mania, depression and quality of life, are described in Table 3.

Regarding circadian rhythm scales, the Composite Scale of Morningness (CSM) was negatively correlated with the appetite dimension, indicating that a higher seasonality of appetite was associated with a later circadian rhythm (evening type). The languid/vigorous factor of the circadian type inventory (CTI), which reflects the physical adaptation to change in circadian rhythm, was positively correlated with appetite and energy seasonality scores, indicating a more languid adaptation (i.e., poorer adaptation) in subjects with a higher seasonality of appetite and energy levels.

The depression and mania scale scores had numerous associations with seasonality scores, indicating that a stronger seasonality was correlated with more severe phenotypes of depression and bipolarity. More specifically, higher scores of manic symptoms, as assessed by the Altman self-rating mania scale (ASRM), were positively correlated with higher seasonality of sleep length and mood, as well as with the psychological factor of the GSS. A higher score on the Quick Inventory of Depressive Symptomatology (QIDS) was positively associated with all seasonality scores and GSS total score.

General health, assessed by the EQ-5D visual analogue scale, was negatively correlated to seasonality of social activities and mood. Looking into the questionnaire’s dimensions, we observed that a higher seasonality of mood was correlated with higher difficulties in self-care, in completing usual activities and anxiety/depression. The pain/discomfort subscale was associated with higher seasonality of sleep length and energy level.

The control versus depressed/bipolar group differences in GSS score are described in Table 4. The GSS psychological factor was associated with diagnosis, and more specifically the sleep length and mood dimensions. The depressed/bipolar group showed a higher seasonality on both of these dimensions (*p* = 0.017 and *p* = 0.006, respectively). However, the food factor and its dimensions were not associated with mood disorder diagnosis. These results are thus very similar to those observed regarding the correlations between the GSS dimensions and the scale assessing manic symptoms (ASRM).

Amongst the depressed/bipolar group, 31.2% of the patients met Kasper et al.’s [1] criteria for SAD compared to 4.7% in the control group (*p* < 0.001). Similarly, 50.0% of the depressed/bipolar group met criteria for S-SAD or SAD, compared to 14.9% in the control group (*p* < 0.001). As described in Table 5, subjects with SAD or S-SAD had a lower score on the CSM and a higher score on the “languid-vigorous” factor of the CTI, indicating that they more frequently presented an evening type and were more languid when facing unusual schedules than subjects with no SAD nor S-SAD. Subjects with SAD or S-SAD also had a higher score on the ASRM and QIDS, indicating higher mania and depression symptoms. Interestingly, it was the subjects with summer SAD/S-SAD who had the latest chronotype, whereas the subjects with more typical winter SAD/S-SAD showed the highest depressive symptoms (Table 6).

There was also a higher rate of subjects declaring that seasonal changes were a problem within the depression/bipolarity group compared to controls (50.0% vs. 31.2%, X^2^ = 4.66, *p*-value = 0.031).

### 3.3. Monthly Variation of the GSS

**Figure 1:**
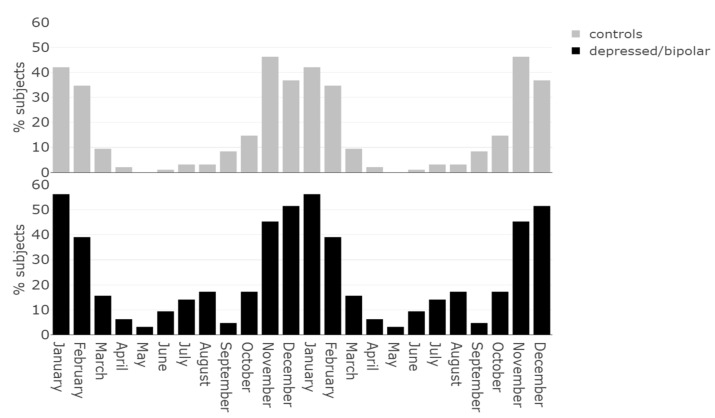
Prevalence of subjects declaring “feeling worst” by month for the control group and depressed/bipolar group. The data was double-plotted to facilitate visual interpretation.

Figure 1 represents the distribution of months where subjects were “feeling worst” for the control and depressed/bipolar groups.

**Figure 2:**
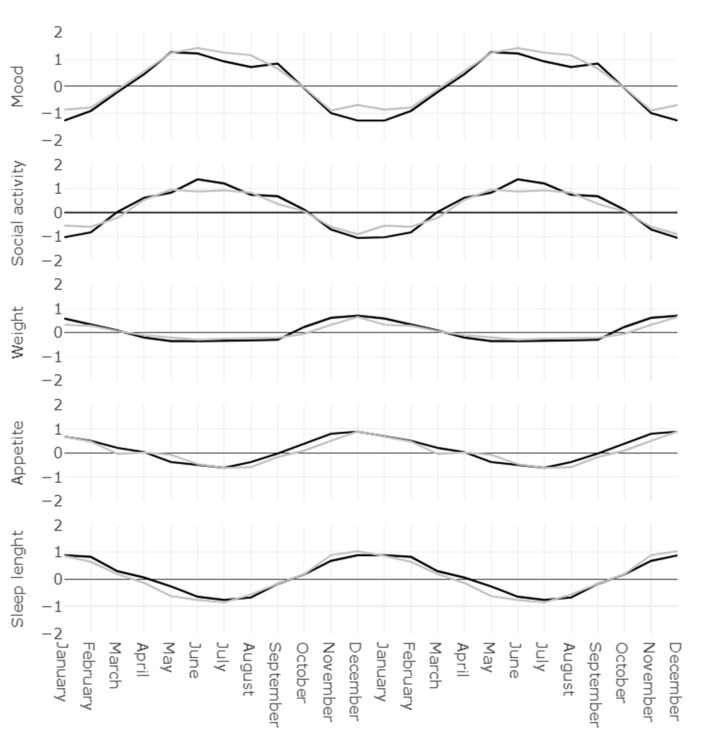
Seasonality scores per months of the GSS dimensions. In gray the control group, in black the depressed/bipolar group. The data was double-plotted to facilitate visual interpretation.

Figure 2 represents the seasonality scores per months of the GSS dimensions. They reflect which months are considered “better” or “worse” on average by the groups, regarding sleep length, social activities, mood, weight and appetite. The mixed models for repeated measure showed that all seasonality scores varied significantly according to month (F_(1 1,1386)_, *p* < 0.001). The group difference between controls and depressed/bipolar subjects was only significant for the mood seasonality score (F_(1,126)_ = 5.67, *p* = 0.019), with a higher seasonality for the mood dimension in the depressed/bipolar group. No other interactions were found between time and group, indicating that the differences between groups did not vary significantly according to month.

### 3.4. Qualifying Seasonality

When using the “feeling best” calendar item to define the season, 13.3% of the subjects declared having a seasonal pattern that could not be defined as being of summer or of winter. However, only 7.8% of the subjects were undefined when using the “feeling worst” calendar item, which was thus retained here. In the depressed/bipolar group, 11.9% (*n* = 7) felt the worst in summer, 69.5 % (*n* = 41) felt the worst in winter and 18.6% (*n* = 11) did not report a seasonal pattern regarding this item. In the control group, none felt the worst in summer, 78% (*n* = 46) felt the worst in winter and 22% (*n* = 13) did not consider feeling the worst according to the season. Thus, simply feeling the worst in summer (regardless of the degree of seasonality and if this was considered as a problem) was specific of the depressed/bipolar group, unlike feeling worst in winter which was common in both groups. Difference between group was significant (*p* = 0.024).

When using the definition of Kasper et al. [1] for SAD and S-SAD for the depressed/bipolar group, 18.8% of patients had a mixed SAD or S-SAD (both winter and summer), 7.8% had a summer SAD or S-SAD, 32.8% had a winter SAD or S-SAD and 40.6% had no SAD or S-SAD. In the control group, 26.6% had a mixed SAD or S-SAD, none had a summer SAD or S-SAD, 10.9% had a winter SAD or S-SAD and 62.5% did not have SAD or S-SAD (*p* = 0.001).

## 4. Discussion

To the best of our knowledge, this is the first study to validate the French version of the SPAQ. All dimensions of the SPAQ, namely, mood, sleep length, energy, social activity, weight and appetite followed seasonal variations. Higher scores of seasonality were associated with worse symptoms of depression, mania and anxiety as well as a later circadian rhythm.

The confirmatory factor analyses of this study further validates the two-factor structure of the SPAQ proposed by Mersch et al. [35], a psychological factor (mood, sleep length, energy and social activity) and a food factor (weight and appetite), with a good fit found by all indicators. Unlike the food factor, the psychological factor was associated with lower quality of life and higher manic symptoms. Together, these results suggest that unlike common practice, the two factors of the GSS should be considered separately, and not as a single total score. However, a single factor structure was found by Young et al. [48]. This discrepancy could be due to characteristics of their sample, as Young et al. only included college students with no previous record of depression or bipolarity, whereas our sample and Mersch et al.’s [35] included patients with SAD, non-seasonal depression and controls. In their study of the seasonality of affective disorder in Switzerland (using a representative sample), Wirz-Justice et al. [49] proposed to divide the GSS into two functional categories, vegetative symptoms (appetite, body weight and sleep duration) and psychological symptoms (mood, social activity and energy change); thus diverging from the factor structure only for sleep. Interestingly, the sleep dimension had the highest loading on the food factor within the psychological factors’ dimensions in both our study and Mersch et al.’s study [35], thus our results are similar to the functional categories proposed.

Our results regarding mixed models for repeated measures indicated that all dimensions of the GSS varied significantly across the year, especially the mood and social activity dimensions. This variation was significantly higher in the depressed/bipolar group compared to controls for the mood dimension. This result is very similar to those found by Mersch et al. [35], with a greater yearly variation of mood and social activity found in depressed patients compared to controls. In our study, both depressed/bipolar and control subjects declared feeling the worst in winter in similar proportions (respectively, 70 and 78%). However, when using Kasper et al.’s [1] criteria for SAD—which includes the degree of variation as well as the subjects’ perceived problem regarding his/her seasonality—we observed significant differences between groups (respectively, 33% and 11%). These results are in line with the idea that “humans are seasonal animals” [50] and it is the extreme variations and their repercussion on daily life which characterizes the SAD [7,8,9].

In our study, both questionnaires and depression/bipolarity diagnosis suggested that marked seasonal patterns were associated with more severe phenotypes of depression and mania, especially regarding the psychological factor and to a lower extent the food factor of the GSS. Similarly, SAD and sub-SAD subjects, according to the Kasper et al.’s definition [1], had higher depression and mania scores. Interestingly, we observed that subjects with higher seasonality scores also differed regarding their circadian rhythm, being more evening types and more languid when facing unusual schedules. This circadian rhythm difference was however only found using the Kasper et al.’s cut-off, and not in the correlation analyses using GSS score, suggesting that circadian rhythm is affected only in severe cases. Those results are consistent with Teicher et al.’s [51] study, reporting a 50 min phase delay in SAD patients using actigraphy as a circadian marker of the rest/activity cycle.

Our results are in line with a growing branch of the literature reporting a seasonal pattern of the severity of symptoms in psychiatric disorders, including hospital admission for mania [52] and major depressive episodes [53]. Young and Dulcis, in their review [54], also describe the seasonal and adjoined photoperiod change as one of the major triggers of switching between mood states in bipolar disorder.

Our study presents several limitations. The first one is the relatively small sample size, which was insufficient for some of the analyses. Indeed, only five subjects met Kasper’s et al.’s criteria [1] for summer SAD or S-SAD, thus tendencies in our results suggesting higher manic symptoms and an earlier circadian rhythm in this population were inconclusive. Furthermore, since the study design does not ensure obtaining a representative sample, prevalence across age and sex could not be studied. Gender differences similar to those found in non-seasonal depression have been reported, with 1.5 times more SAD in women than in men [15]. However, SAD seems to regress with age [15] whereas non-seasonal depression prevalences are relatively stable past age 30 [55]. Another limitation is the lack of clinically-based diagnosis for SAD, which we thus based, as most studies on the subject, on Kasper et al.’s criteria. Thus, we could not verify if these criteria showed good fit in our sample, which could have been of interest considering the wide range in sensitivity and specificity reported in the literature. Finally, the cross-sectional aspect of our study does not allow for conclusion regarding the directionality of associations.

Several physiological mechanisms have been suggested to be involved in the relationship between mood and seasonality. Light synchronizes the master clock located in the suprachiasmatic nuclei (SCN) to the external day-night cycle, and this central pacemaker in turn synchronizes all biological circadian rhythms [17]. Light information reaches the SCN through the retinohypothalamic tract (RHT) and the photoperiod—which can be defined as the day length—regulates the expression levels of the core clock genes (CCG), thereby enabling the SCN to encode for day length variability and thus seasonal changes [17]. This rhythmic expression of CCG drives many physiological functions, including the sleep-wake cycle, mood regulation, diet, thermoregulation and hormonal secretion, as well as lipid and carbohydrate metabolism [17,56,57]. This central rhythmic information is relayed to other parts of the brain, including the pineal gland where the activation of the transcription factors, AA-NAT and ASMT, which drive the photoperiodic synthesis and nocturnal release of melatonin, which in turn also regulate other peripheral oscillators [17,58]. Adaptation to seasonal photoperiodic change, including change in the secretion pattern of melatonin, could be altered in SAD patients and desynchronize endogenous rhythms and alter sleep architecture. It has been observed that melatonin secretion lasts longer in winter than in summer in patients with SAD [17,59]. The importance of the photoperiod and adaptation to seasons has been shown in a primate model of SAD where monkeys displayed depression-related behavioral and physiological changes in response to short photoperiod conditions which could be reversed by antidepressant treatment [60]. This difficulty in adapting to seasonal photoperiodic change could be explained by a potential alteration in the melanopsin system and light sensitivity in SAD patients [61].

Mood is one domain largely recognized to be affected by season, going from the general population with subsyndromic symptoms to individuals diagnosed with mood disorders [8]. Seasons also impact cognitive brain functions, neuroendocrine function, reproduction and many other physiological domains [50]. Several studies using post-mortem brain, cerebral spinal fluid, peripheral biochemical markers, but also more recent imaging studies, show seasonal changes in brain monoamine systems, such as serotonin and dopamine [50]. The duration of bright sunlight has also a direct impact on serotonin turnover in the brain [62].

Light also has a direct effect on monoaminergic pathways through a SCN-independent pathway linking the retina to mood regulation areas, such as the perihabenular nucleus [63,64]. These direct actions on monoaminergic systems may explain the rapid antidepressant effects of light therapy (LT) of about 2–3 days [65,66,67]. Light also activates efferent serotonergic neurons, to decrease the serotonin reuptake transporter (5-HTT) levels and increase serotonin (5-HT) levels in mood regulatory areas such as the anterior cingulate and prefrontal cortex [25,66,68]. Light also acts on the sleep homeostasis process by increasing the sleep intensity [69]. In addition, it has been demonstrated that LT increases alertness [70], including after a night of sleep deprivation and a morning exposition to LT [71]. LT has also been shown to improve sleepiness and sustained attention, among other cognitive functions [67,71].

Overall, our results indicate that the French version of the SPAQ is both valid and reliable, with a two-factor structure. Mood and social activity dimensions are significantly affected by seasons in depressed/bipolar patients and a stronger seasonality is associated with more severe phenotypes of depression and mania. Simple tools to better qualify seasonal changes, such as the SPAQ, can have direct implications on the therapeutics of mood disorders, including the use of bright light therapy in order to enhance personalized treatments and prevent adverse seasonal effects [72,73].

## Figures and Tables

**Table 1:** Pearson correlation matrix of the general seasonality score (GSS) dimensions.

	Sleep Length	Social Activities	Mood	Energy Level	Weight	Appetite
Sleep length	—					
Social activities	0.379 ***	—				
Mood	0.318 ***	0.536 ***	—			
Energy level	0.432 ***	0.560 ***	0.663 ***	—		
Weight	0.322 ***	0.269 ***	0.246 ***	0.408 ***	—	
Appetite	0.301 ***	0.270 ***	0.335 ***	0.441 ***	0.698 ***	—

GSS: general seasonality score of the Seasonal Pattern Assessment Questionnaire (SPAQ). *** *p* < 0.001.

**Table 2:** Factor loading of the confirmatory factor analysis (CFA) of the GSS.

Factor	Dimension	Estimate	SE	Z	*p*
Psychological factor	Sleep length	0.542	0.086	6.332	<0.001
	Social activities	0.714	0.081	8.783	<0.001
	Mood	0.860	0.082	10.431	<0.001
	Energy level	0.968	0.076	12.680	<0.001
Food factor	Weight	0.995	0.094	10.562	<0.001
	Appetite	0.872	0.090	9.665	<0.001

GSS: general seasonality score of the Seasonal Pattern Assessment Questionnaire (SPAQ).

**Table 3:** Kendall’s tau correlation between the GSS (dimensions, factors and total score) and questionnaires.

	CSM-Chronotype	CTI FR-Flexibility/Rigidity Factor	CTI LV-Languid/Vigorous Factor	ASRM-Manic Symptoms	QIDS-Depressive Symptoms	EQ5D 1-Mobility	EQ5D 2-Self-Care	EQ5D 3-Usual Activities	EQ5D 4-Pain/Discomfort	EQ5D 5-Anxiety/Depression	EQ5D VAS-Global Health
**Psychological factor**	−0.09	0.01	0.12	**0.15**	**0.26**	0.08	0.08	0.14	0.14	0.19	**−0.18**
Sleep length	−0.10	−0.04	0.10	**0.19**	**0.19**	0.10	0.00	0.12	**0.18**	0.07	−0.13
Social activities	−0.03	0.03	0.06	0.03	**0.15**	0.04	0.09	0.09	0.00	0.15	**−0.19**
Mood	−0.04	0.01	0.08	**0.16**	**0.28**	0.06	**0.16**	**0.15**	0.14	**0.28**	**−0.19**
Energy level	−0.08	0.02	**0.15**	0.12	**0.25**	0.08	0.04	0.13	**0.16**	0.14	−0.12
**Food factor**	−0.12	−0.01	**0.16**	0.13	**0.17**	−0.09	0.00	0.04	0.00	0.03	−0.04
Weight	−0.10	−0.04	0.13	0.14	**0.17**	−0.11	−0.05	0.03	0.00	0.04	−0.09
Appetite	**−0.15**	0.02	**0.18**	0.12	**0.15**	−0.06	0.04	0.03	−0.02	0.01	0.00
**GSS total**	−0.10	0.00	**0.13**	**0.15**	**0.26**	0.04	0.07	0.12	0.10	**0.15**	**−0.15**

GSS: general seasonality score of the Seasonal Pattern Assessment Questionnaire (SPAQ). Psychological factor: GSS factor derived from the confirmatory analysis combining the dimensions of sleep length, social activities, mood and energy level. Food factor: GSS factor derived from the confirmatory analysis combining the dimensions of weight and appetite (higher score means more seasonality). CSM, Composite Scale of Morningness (higher score means earlier chronotype). CTI FR: circadian type inventory flexibility/rigidity factor, CTI LV: circadian type inventory languid/vigorous factor (higher score means more flexible and more languid, respectively). ASRM: Altman self-rating mania scale (higher score means greater manic symptoms). QIDS: Quick Inventory of Depressive Symptomatology (higher score means greater depressive symptoms). EQ-5D: European Quality of life-5 Dimension. EQ-5D 1–5 (higher score means more difficulties), mobility, self-care, usual activities, pain/discomfort, anxiety/depression. EQ-5D VAS, visual analog scale of global health (higher score means better health). Bold correlations are those with a *p*-values < 0.05.

**Table 4:** Difference in GSS between controls and patients with a diagnosis of depression or bipolarity (Mann–Whitney U test).

	Depressed/Bipolar (*n* = 64) Mean (SD)	Control (*n* = 64) Mean (SD)	*p*
Psychological factor	8.33 (3.62)	6.73 (3.29)	**0.013**
Sleep length	1.80 (1.21)	1.28 (1.03)	**0.017**
Social activities	2.00 (1.10)	1.70 (1.05)	0.118
Mood	2.27 (1.19)	1.73 (1.04)	**0.006**
Energy level	2.27 (1.17)	2.02 (1.12)	0.187
Food factor	2.75 (2.29)	2.13 (1.78)	0.144
Weight	1.27 (1.24)	0.94 (0.96)	0.195
Appetite	1.48 (1.22)	1.19 (1.04)	0.184
GSS total	11.08 (5.05)	8.86 (4.57)	**0.013**

GSS: general seasonality score of the Seasonal Pattern Assessment Questionnaire (SPAQ). Psychological factor: GSS factor derived from the confirmatory analysis combining the dimensions of sleep length, social activities, mood and energy level. Food factor: SPAQ GSS factor derived from the confirmatory analysis combining the dimensions of weight and appetite. Bold associations are those with a *p*-values < 0.05. SD: Standard Deviation

**Table 5:** Difference in circadian rhythm, mania and depression scores between SAD, S-SAD and non-SAD subjects, according to Kasper et al.’s definition.

	No SAD (*n* = 23)Mean (SD)	SAD (*n* = 105)Mean (SD)	*p* ^a^	No SAD or S-SAD (*n* = 66), Mean (SD)	SAD or S-SAD (*n* = 62), Mean (SD)	*p* ^b^
CSM	35.92 (7.67)	34.61 (7.68)	0.479	37.02 (38.00)	34.27 (34.00)	**0.025**
CTI_FR	12.58 (4.61)	12.22 (4.00)	0.779	12.29 (11.00)	12.76 (13.00)	0.436
CTI_LV	19.42 (4.85)	20.61 (4.69)	0.245	18.79 (19.00)	20.53 (21.00)	**0.039**
ASRM	2.05 (2.82)	3.83 (4.64)	0.062	1.80 (0.00)	2.97 (1.00)	**0.050**
QIDS	5.42 (4.49)	10.17 (6.10)	**<0.001**	5.33 (3.50)	7.27 (6.00)	**0.004**

SAD: seasonal affective disorder. S-SAD: subsyndromal-SAD. CSM, Composite Scale of Morningness (higher score means earlier chronotype). CTI FR: circadian type inventory flexibility/rigidity factor, CTI LV: circadian type inventory languid/vigorous factor (higher score means more flexible and more languid, respectively). ASRM: Altman self-rating mania scale (higher score means greater manic symptoms). QIDS: Quick Inventory of Depressive Symptomatology (higher score means greater depressive symptoms). ^a^ Mann–Whitney U test difference between SAD and non-SAD. ^b^ Mann–Whitney U test difference between SAD/S-SAD and non-SAD/S-SAD. Bold associations are those with a *p*-values < 0.05. SD: Standard Deviation

**Table 6:** Difference in circadian rhythm, mania and depression scores between winter, summer or mixed SAD or S-SAD and non-SAD or S-SAD subjects, according to Kasper et al.’s definition (Kruskal–Wallis test).

	No SAD or S-SAD (*n* = 66)	Winter SAD or S-SAD (*n* = 28)	Summer SAD or S-SAD (*n* = 5)	Mixed SAD or S-SAD (*n* = 29)	*p*
CSM	37.0 (7.67)	36.7 (5.82)	30.2 (9.93)	32.7 (7.92)	**0.044**
CTI_FR	12.3 (4.72)	12.3 (3.96)	15.4 (6.15)	12.7 (4.20)	0.447
CTI_LV	18.8 (5.10)	19.5 (4.54)	24.0 (1.87)	21.0 (4.21)	0.119
ASRM	1.80 (2.61)	3.00 (3.94)	5.20 (5.07)	2.55 (3.40)	0.253
QIDS	5.33 (5.08)	8.32 (5.56)	6.00 (4.74)	6.48 (4.49)	**0.004**

SAD: seasonal affective disorder. S-SAD: subsyndromal-SAD. Bold associations are those with a *p*-values < 0.05.

## Data Availability

The data is not available.

## Supplementary Materials

The following are available online at https://www.mdpi.com/article/10.3390/jcm10091897/s1, File S1: French version of the Seasonal Pattern Assessment Questionnaire (SPAQ); Figure S1: Path diagram of the confirmatory factor analysis of the general seasonality score (Seasonal Pattern Assessment Questionnaire – SPAQ).
