# Supplementary material for: Validity and Usage of the Seasonal Pattern Assessment Questionnaire (SPAQ) in a French Population of Patients with Depression, Bipolar Disorders and Controls
Source: J Clin Med. 2021 Apr 27;10(9):1897. doi: 10.3390/jcm10091897 (PMC8123881; doi:10.3390/jcm10091897)
Supplement: Supplementary file 1 [file jcm-10-01897-s001.zip › jcm-1164635-supplementary/Figure S1.pdf]

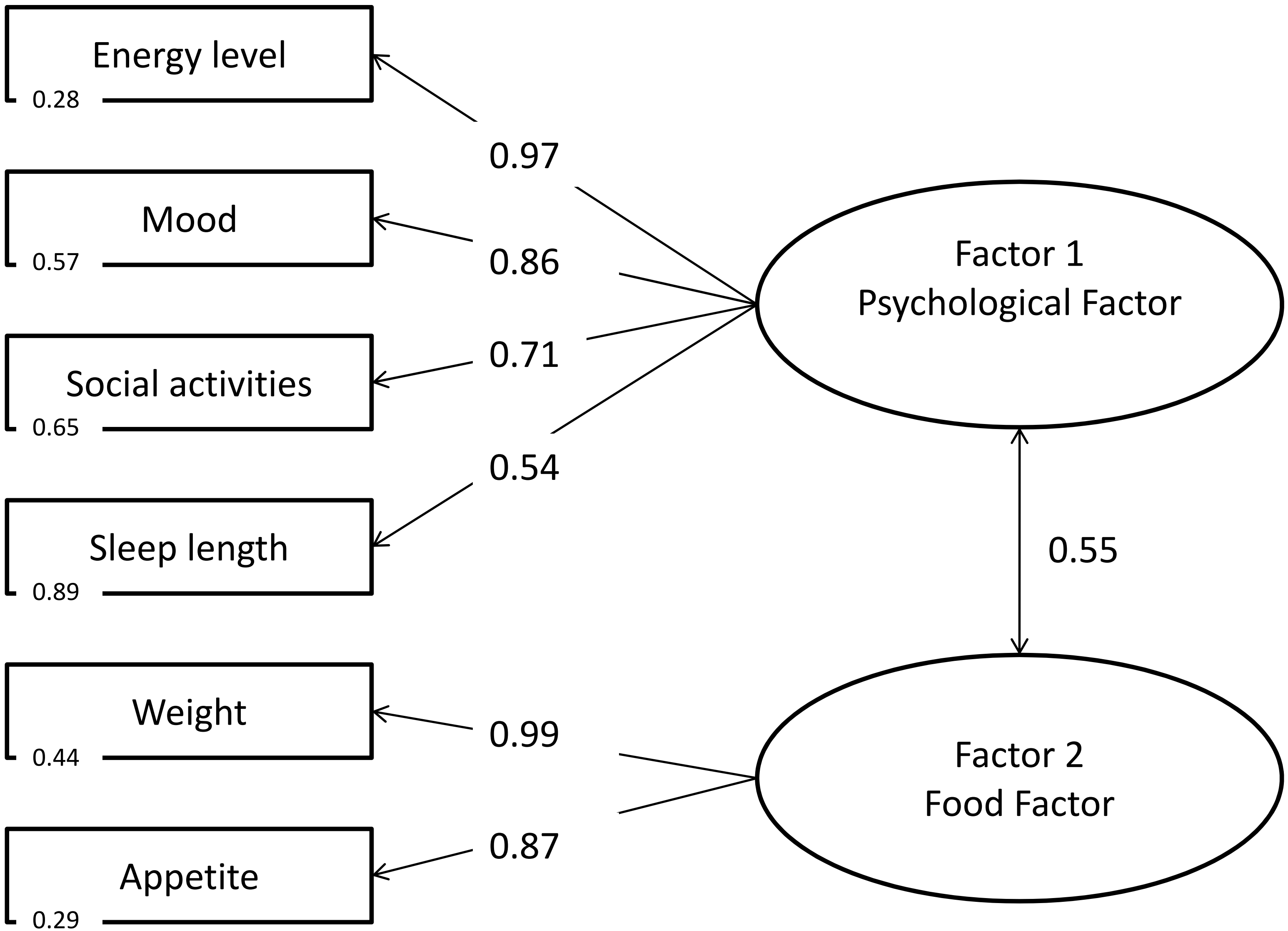


**Figure S1.** Path diagram of the confirmatory factor analysis of the General Seasonality Score (Seasonal Pattern Assessment Questionnaire – SPAQ).

The single-headed arrows are the factor loadings of each dimension, the double-headed arrow is the covariance between the two factors and the number assigned to each dimension is the corresponding residual covariance.
