# Supplementary material for: Validity and Usage of the Seasonal Pattern Assessment Questionnaire (SPAQ) in a French Population of Patients with Depression, Bipolar Disorders and Controls
Source: J Clin Med. 2021 Apr 27;10(9):1897. doi: 10.3390/jcm10091897 (PMC8123881; doi:10.3390/jcm10091897)
Supplement: Supplementary file 1 [file jcm-10-01897-s001.zip › jcm-1164635-supplementary/File S1.pdf]

## File S1-French version of the Seasonal Pattern Assessment Questionnaire (SPAQ)

## QUESTIONNAIRE D'ÉVALUATION DU PROFIL SAISONNIER*

**1. Nom** ________________________________ **2. Age** ____________

**3. Lieu de naissance – ville / région / pays** ________________________________

**4. Date du jour** ______ ______ ______

Jour Mois Année

**INSTRUCTIONS**

*** Entourer la bonne réponse**

**Exemple :**

**Sexe Masculin 1 Féminin 2**

**5. Poids corporel (Kg)** ____________

**6. Années d'éducation**

| | |
|---|---|
| Moins de 4 ans de lycée | 1 |
| Lycée seulement | 2 |
| 1 – 3 ans après le lycée | 3 |
| 4 ans ou plus après le lycée | 4 |

**7. Sexe** Masculin 1 Féminin 2

**8. Statut marital**

| | |
|---|---|
| Célibataire | 1 |
| Marié | 2 |
| Séparé/Divorcé | 3 |
| Veuf(ve) | 4 |

**9. Activité professionnelle** ________________________________

**10. Depuis combien d'années vivez-vous dans cette région climatique ?** ________________________________

**L'objectif de ce questionnaire est d'évaluer les modifications de votre humeur et de votre comportement à travers le temps.**
**Remplissez s'il vous plaît tous les champs.**
**Note: Nous sommes intéressés par votre expérience; pas par ce que d'autres ont pu observer chez vous.**

**11. Indiquez le degré de changement selon les saisons ?**

| | Pas de changement | Changement léger | Changement modéré | Changement prononcé | Changement très prononcé |
|---|---|---|---|---|---|
| A. Durée du sommeil | 0 | 1 | 2 | 3 | 4 |
| B. Activité sociale | 0 | 1 | 2 | 3 | 4 |
| C. Humeur (sensation de bien-être) | 0 | 1 | 2 | 3 | 4 |
| D. Poids | 0 | 1 | 2 | 3 | 4 |
| E. Appétit | 0 | 1 | 2 | 3 | 4 |
| F. Niveau d'énergie | 0 | 1 | 2 | 3 | 4 |

12. Pour les questions suivantes, remplissez les cercles lorsque la condition s'applique pour les mois concernés. Cela peut être un seul mois O, un groupe de mois, i.e O O O, ou toute autre combinaison de mois.

| A quel moment de l'année ... | JAN | FEV | MAR | AVR | MAI | JUIN | JUIL | AOUT | SEPT | OCT | NOV | DEC | Ou Pas de mois particulier |
|---|---|---|---|---|---|---|---|---|---|---|---|---|---|
| A. Vous sentez-vous le mieux | O | O | O | O | O | O | O | O | O | O | O | O | O |
| B. Prenez vous le plus de poids | O | O | O | O | O | O | O | O | O | O | O | O | O |
| C. Êtes-vous le plus sociable | O | O | O | O | O | O | O | O | O | O | O | O | O |
| D. Dormez-vous le moins | O | O | O | O | O | O | O | O | O | O | O | O | O |
| E. Mangez-vous le plus | O | O | O | O | O | O | O | O | O | O | O | O | O |
| F. Perdez-vous le plus de poids | O | O | O | O | O | O | O | O | O | O | O | O | O |
| G. Êtes-vous le moins sociable | O | O | O | O | O | O | O | O | O | O | O | O | O |
| H. Vous sentez-vous le moins bien | O | O | O | O | O | O | O | O | O | O | O | O | O |
| I. Mangez-vous le moins | O | O | O | O | O | O | O | O | O | O | O | O | O |
| J. Dormez-vous le plus | O | O | O | O | O | O | O | O | O | O | O | O | O |

13. De combien votre poids fluctue t'il au cours de l'année ?

| | | | |
|---|---|---|---|
| 0-2 kg | 1 | 6-8 kg | 4 |
| 2-4 kg | 2 | 8-10 kg | 5 |
| 4-6 kg | 3 | > 10 | 6 |

14. Approximativement combien d'heures dormez-vous par journée de 24 heures pendant chaque saison ? (en incluant les siestes)

| | | | | | | | | | | | | | | | | | | | | |
|---|---|---|---|---|---|---|---|---|---|---|---|---|---|---|---|---|---|---|---|---|
| Hiver | 0 | 1 | 2 | 3 | 4 | 5 | 6 | 7 | 8 | 9 | 10 | 11 | 12 | 13 | 14 | 15 | 16 | 17 | 18 | Plus de 18 |
| Printemps | 0 | 1 | 2 | 3 | 4 | 5 | 6 | 7 | 8 | 9 | 10 | 11 | 12 | 13 | 14 | 15 | 16 | 17 | 18 | Plus de 18 |
| Été | 0 | 1 | 2 | 3 | 4 | 5 | 6 | 7 | 8 | 9 | 10 | 11 | 12 | 13 | 14 | 15 | 16 | 17 | 18 | Plus de 18 |
| Automne | 0 | 1 | 2 | 3 | 4 | 5 | 6 | 7 | 8 | 9 | 10 | 11 | 12 | 13 | 14 | 15 | 16 | 17 | 18 | Plus de 18 |

15. Ressentez-vous une modification de vos préférences alimentaires selon les différentes saisons ?

Non 1 Oui 2 Si oui, précisez :

16. Si vous ressentez des changements avec les saisons, estimez-vous qu'ils sont un problème pour vous ?

Non 1 Oui 2 Si oui, ce problème est :

| | |
|---|---|
| Léger | 1 |
| Modéré | 2 |
| Marqué | 3 |
| Sévère | 4 |
| Handicapant | 5 |

*Traduit par PA Geoffroy & E Haffen, d'après Rosenthal et al 1987.

# SEASONAL PATTERN ASSESSMENT QUESTIONNAIRE

**1. Name** ____________________ **2. Age** ____________

**3. Place of birth – City / Province (State) / Country** ____________________

**4. Today's date** ______ ______ ______

Month Day Year

**INSTRUCTIONS**

*** Please circle the number beside your choice**

**Example:**

**Sex Male 1 Female 2**

**5. Current weight (in lbs.)** ____________

| | | |
|---|---|---|
| **6. Years of education** | **Less than four years of high school** | **1** |
| | **High school only** | **2** |
| | **1 – 3 years post high school** | **3** |
| | **4 or more years post high school** | **4** |
| **7. Sex** | **Male 1** | **Female 2** |
| **8. Marital Status** | **Single** | **1** |
| | **Married** | **2** |
| | **Separated/Divorced** | **3** |
| | **Widowed** | **4** |

**9. Occupation** ____________________

**10. How many years have you lived in this climatic area?** ____________________

**The purpose of this for is to find out how your mood and behaviour change over time. Please fill in all the relevant circles. Note: We are interested in your experience; not others you may have observed**

**11. To what degree do the following change with the seasons?**

| | No change | Slight change | Moderate change | Marked change | Extremely marked change |
|---|---|---|---|---|---|
| A. Sleep length | 0 | 1 | 2 | 3 | 4 |
| B. Social activity | 0 | 1 | 2 | 3 | 4 |
| C. Mood (overall feeling of wellbeing) | 0 | 1 | 2 | 3 | 4 |
| D. Weight | 0 | 1 | 2 | 3 | 4 |
| E. Appetite | 0 | 1 | 2 | 3 | 4 |
| F. Energy Level | 0 | 1 | 2 | 3 | 4 |

12. In the following questions, fill in circles for all applicable months. This may be a single month, O, a cluster of months, e.g. O O, or any other grouping.

At what time of the year do you…

| | JAN | FEB | MAR | APR | MAY | JUN | JUL | AUG | SEP | OCT | NOV | DEC | OR | No particular month stand out as extreme |
|---|---|---|---|---|---|---|---|---|---|---|---|---|---|---|
| A. Feel best | O | O | O | O | O | O | O | O | O | O | O | O | | O |
| B. Gain most weight | O | O | O | O | O | O | O | O | O | O | O | O | | O |
| C. Socialize most | O | O | O | O | O | O | O | O | O | O | O | O | | O |
| D. Sleep least | O | O | O | O | O | O | O | O | O | O | O | O | | O |
| E. Eat most | O | O | O | O | O | O | O | O | O | O | O | O | | O |
| F. Lose most weight | O | O | O | O | O | O | O | O | O | O | O | O | | O |
| G. Socialize least | O | O | O | O | O | O | O | O | O | O | O | O | | O |
| H. Feel worst | O | O | O | O | O | O | O | O | O | O | O | O | | O |
| I. Eat least | O | O | O | O | O | O | O | O | O | O | O | O | | O |
| J. Sleep most | O | O | O | O | O | O | O | O | O | O | O | O | | O |

13. How much does your weight fluctuate during the course of the year?

| | | | |
|---|---|---|---|
| 0-3 lbs | 1 | 12-15 lbs | 4 |
| 4-7 lbs | 2 | 16-20 lbs | 5 |
| 8-11 lbs | 3 | > 20 lbs | 6 |

14. Approximately how many hours of each 24-hour day do you sleep during each season? (Include naps)

| | | | | | | | | | | | | | | | | | | | | |
|---|---|---|---|---|---|---|---|---|---|---|---|---|---|---|---|---|---|---|---|---|
| Winter | 0 | 1 | 2 | 3 | 4 | 5 | 6 | 7 | 8 | 9 | 10 | 11 | 12 | 13 | 14 | 15 | 16 | 17 | 18 | Over 18 |
| Spring | 0 | 1 | 2 | 3 | 4 | 5 | 6 | 7 | 8 | 9 | 10 | 11 | 12 | 13 | 14 | 15 | 16 | 17 | 18 | Over 18 |
| Summer | 0 | 1 | 2 | 3 | 4 | 5 | 6 | 7 | 8 | 9 | 10 | 11 | 12 | 13 | 14 | 15 | 16 | 17 | 18 | Over 18 |
| Fall | 0 | 1 | 2 | 3 | 4 | 5 | 6 | 7 | 8 | 9 | 10 | 11 | 12 | 13 | 14 | 15 | 16 | 17 | 18 | Over 18 |

15. Do you notice a change in food preference during the different seasons?

No 1 Yes 2 If yes, please specify:

16. If you experience changes with the seasons, do you feel that these are a problem for you?

No 1 Yes 2 If yes, is this problem -

| | |
|---|---|
| Mild | 1 |
| Moderate | 2 |
| Marked | 3 |
| Severe | 4 |
| Disabling | 5 |
